# *Bacillus thuringiensis* Cry5B Protein Is Highly Efficacious as a Single-Dose Therapy against an Intestinal Roundworm Infection in Mice

**DOI:** 10.1371/journal.pntd.0000614

**Published:** 2010-03-02

**Authors:** Yan Hu, Sophia B. Georghiou, Alan J. Kelleher, Raffi V. Aroian

**Affiliations:** Section of Cell and Developmental Biology, University of California, San Diego, La Jolla, California, United States of America; Swiss Tropical Institute, Switzerland

## Abstract

**Background:**

Intestinal parasitic nematode diseases are one of the great diseases of our time. Intestinal roundworm parasites, including hookworms, whipworms, and *Ascaris*, infect well over 1 billion people and cause significant morbidity, especially in children and pregnant women. To date, there is only one drug, albendazole, with adequate efficacy against these parasites to be used in mass drug administration, although tribendimidine may emerge as a second. Given the hundreds of millions of people to be treated, the threat of parasite resistance, and the inadequacy of current treatments, new anthelmintics are urgently needed. *Bacillus thuringiensis* (Bt) crystal (Cry) proteins are the most common used biologically produced insecticides in the world and are considered non-toxic to vertebrates.

**Methods/Principal Findings:**

Here we study the ability of a nematicidal Cry protein, Cry5B, to effect a cure in mice of a chronic roundworm infection caused by the natural intestinal parasite, *Heligmosomoides bakeri* (formerly *polygyrus*). We show that Cry5B produced from either of two Bt strains can act as an anthelmintic *in vivo* when administered as a single dose, achieving a ∼98% reduction in parasite egg production and ∼70% reduction in worm burdens when delivered *per os* at ∼700 nmoles/kg (90–100 mg/kg). Furthermore, our data, combined with the findings of others, suggest that the relative efficacy of Cry5B is either comparable or superior to current anthelmintics. We also demonstrate that Cry5B is likely to be degraded quite rapidly in the stomach, suggesting that the actual dose reaching the parasites is very small.

**Conclusions/Significance:**

This study indicates that Bt Cry proteins such as Cry5B have excellent anthelmintic properties *in vivo* and that proper formulation of the protein is likely to reveal a superior anthelmintic.

## Author Summary

Intestinal parasitic nematode diseases infect over one billion people and cause significant disease burden in children (growth and cognitive stunting, malnutrition), in pregnant women, and via their dampening of the immune system in infected individuals. In over thirty years, no new classes of anti-roundworm drugs (anthelmintics) for treating humans have been developed. Because of limitations of the current drugs and the threat of parasite resistance, new anthelmintics are needed. The soil bacterium *Bacillus thuringiensis* (Bt) produces crystal (Cry) proteins that specifically target and kill insects and nematodes and is used around the world as a safe insecticide. Here we test the effects of the Bt Cry protein Cry5B on a chronic, natural intestinal roundworm infection in mice, namely the helminth parasite *Heligmosomoides bakeri*. We find that a single dose of Cry5B can eliminate 70% of the parasites and can almost completely block the ability of the parasites to produce progeny. Comparisons of Cry5B's efficacy with known anthelmintics suggest its activity is as good as or perhaps even better than those currently used. Furthermore, this protein is rapidly digested by simulated stomach juices, suggesting that protecting it from these juices would reveal a superior anthelmintic.

## Introduction

Neglected tropical diseases (NTDs) have a worldwide devastating impact on the lives of billions of people. Helminth infections comprise approximately 85% of the NTD burden [1]. The top three ailments on this list of NTDs are all caused by intestinal nematodes [2]. These infections consist of ascariasis (caused by *Ascaris lumbricoides*), trichuriasis (caused by *Trichuris trichiura* or whipworm), and hookworm disease (caused by *Necator americanus* and *Ancylostoma duodenale*). Approximately 807-1,221 million people are afflicted with ascariasis, 604–795 million with trichuriasis, and 576–740 million with hookworm infections [3]. The widespread and detrimental effects of parasitic worm infections on human growth, nutrition, cognition, school attendance and performance, earnings, and pregnancy have been well documented [2],[3]. These infections also contribute to increased severity/infectivity of HIV/AIDS, malaria, and tuberculosis due to compromised immune responses [3],[4]. Furthermore, parasitic nematode infections confound vaccination efficacy [5],[6]. Despite the high prevalence and destructive nature of these infections, there are few treatment options. Although four anthelmintics (levamisole/pyrantel and mebendazole/albendazole) are approved by the World Health Organization for use in humans, one, albendazole, is generally preferred in a single-dose regimen over the others since it is relatively more effective against hookworms and whipworms [7],[8]. However, resistance to albendazole may already be appearing [9],[10]. Furthermore, the reliance upon one compound for treating hundreds of millions of people will have devastating consequences if widespread resistance ever becomes a reality. Tribendimidine, developed by the Chinese Centers for Disease Control and Prevention, is emerging as a second anthelmintic with efficacy similar to albendazole, but is a member of the levamisole/pyrantel class to which resistance in human populations has been reported [11],[12],[13]. Furthermore, none of the compounds have been shown to be totally effective against all helminth infections [8]. Consequently, there is an urgent need for efficacious, safe, inexpensive, single-dose anthelmintics with new mechanisms of action.

This search for new anthelmintics has led to examination of *Bacillus thuringiensis* (Bt) crystal (Cry) proteins. These proteins are the most extensively used biologically-produced insecticides in the world [14]. Bt is a soil bacterium that produces crystal inclusions during sporulation. These inclusions contain Cry proteins that are highly toxic to some invertebrates but nontoxic to humans and other vertebrates [15]. The high efficacy against insects, absence of toxicity towards vertebrates, and low production cost of these proteins has led to their widespread use in pesticides and in transgenic crops [14]. So far, three Bt Cry proteins toxic to a broad range of free-living nematodes and the free-living form of at least one intestinal parasitic nematode have been discovered, including: Cry5B, Cry14A, and Cry21A [16]. Cry13A may also have anti-nematode activity [17]. To date, only one of these, Cry5B, has been shown to be therapeutic *in vivo* with activity against intestinal hookworm parasite (*Ancylostoma ceylanicum*) infections in hamsters when delivered daily, *per os*, over the course of three days [18].

These studies suggest that Cry proteins could provide therapy for intestinal nematode infections. However, it remains to be shown that Cry5B can effect a cure against more than *A. ceylanicum* infections in hamsters or that Cry proteins are efficacious as single-dose anthelmintics. *Heligmosomoides bakeri* (formerly known as *Heligmosomoides polygyrus* and *Nematospiroides dubius*) is one of the most widely studied rodent intestinal parasite nematodes [19],[20]. The nematode has a high infection rate and is the best model for chronic intestinal nematode infections in immunocompetent mice. *H. bakeri* has also played a key role in the history of anthelmintic development via its use in the discovery of ivermectin [21]. In addition, *H. bakeri* infections in mice are a naturally occurring infection, unlike *A. ceylanicum* infections in hamsters. Thus, curative experiments in *H. bakeri* are complementary to those in *A. ceylanicum*, yielding important information as to how Cry proteins may fare against a broad range of natural intestinal parasites *in vivo*. Herein, we report our investigations into single-dose Cry5B therapy against *H. bakeri*.

## Methods

### Animals

Female Swiss Webster white mice were purchased from Harlan Laboratories and were infected at approximately 6 weeks of age at an average weight of 25g. Mice were provided with food and water *ad libitum*.). This research was approved by the UCSD Institutional Animal Care and Use Committee (IACUC), protocol number S08140. The maintenance and care of experimental animals complied with the University of California's Animal Care Program's guidelines for the humane use of laboratory animals.

### Preparation of Bt strains

Crystal-deficient Bt strains HD1 and 4Q7 were transformed with a plasmid containing the Cry5B gene [23]. *Bacillus thuringiensis* subspecies *kurstaki* HD1-4D8 was ordered through the *Bacillus* Genetic Stock Center. Spore lysates (SLs; HD1 and 4Q7 Cry-deficient strains) and spore-crystal lysates (SCLs; HD1 and 4Q7 transformed with Cry5B plasmid) were prepared using standard methods and then stored at −80° [23]. Bioactivity of SCLs was confirmed against *Caenorhabditis elegans* by a mortality assay over 24 h at 25°C. SLs (Cry-minus) were confirmed to lack toxicity against *C. elegans*. On the day of use, SL and Cry5B SCL aliquots were thawed and centrifuged at 4,500 rpm for 15 minutes at 4°C and the supernatant was removed. The pellet was then resuspended in distilled water to a final concentration of 2.5 mg/mL, for the HD1 strain, and 2.25 mg/mL, for the 4Q7 strain (protein concentrations were determined by comparing Cry5B band intensities for four different aliquots of SCLs to known amounts of bovine serum albumin on Coomassie-stained 8% SDS polyacrylamide protein gels). The placebo SL control strains were concentrated to the same extent. The samples were kept on ice until gavage.

### Cry5B curative experiments

On day 0, mice were infected *per os* with a suspension of 200±10 *H. bakeri* L3 larvae in 0.1 mL of distilled water. Larvae were counted under the microscope, then drawn into a pipette tip and placed into separate glass test tubes until gavage with a blunt-ended syringe. On days 14, 16, 18, and 20 post-infection (P.I.), fecal samples were collected from the mice. Mice were placed individually in empty plastic cages for 1 h each morning, and the fecal pellets were collected into 50 mL centrifuge tubes. The number of eggs present was counted using the modified McMaster technique [22]. Briefly, feces collected from mice were weighed and resuspended in a 1 g:15 mL volume of water. The pellets were allowed to soak overnight before being broken up for 1 h via heavy vortexing. The eggs were counted using a 2-chamber McMaster slide, each chamber holding a 0.6 mL volume of a 1∶1 mixture of fecal slurry and saturated sucrose solution. The number of eggs per gram of feces was thus calculated from the following equation: number of eggs counted x (1/0.3 mL slurry) x (15 mL slurry/g feces). For each mouse and each time point, three different egg counts were made and then averaged.

Each mouse was treated *per os* on day 15 P.I. with 0.1 mL of relevant treatment (placebo or Cry protein) through a blunt-ended syringe. All mice were killed by exposure to CO_2_ on day 20 P.I. and the intestines were removed in their entirety. These were opened longitudinally with a pair of blunt-ended dissecting scissors and then placed into a 50 mL centrifuge tube with 10–20 mL of pre-warmed (37°C) PBS for approximately 1 h to allow worms to dislodge from the intestine. The solution and intestine were examined under a microscope, using fine tweezers when necessary for further extrication of worms from the intestine, for determination of final worm burden.

### Tribendimidine curative experiments

Tribendimidine was kindly provided by Dr. Shu-Hua Xiao at the Chinese Centers for Disease Control and Prevention. The drug was suspended in 20 mM citrate buffer pH 7.3 and delivered *per os* on day 15 P.I. in a total volume of 0.1 mL as per Cry5B experiments. For these curative experiments, the mice were infected with on average 150 L3 larvae (six/group except for placebo group, which only had five mice). Placebo control for these experiments was 0.1 mL of buffer only.

### Digestive fluids for Cry5B metabolic fate studies

Simulated gastric fluid (SGF) was prepared freshly as described in the United States Pharmacopeia and stored at 4° until use [24]. Cry5B SLC was added to a 1 mL solution of SGF for a final concentration of 2.5 mg/mL and incubated at 37°C [25]. 50 µL aliquots of the digestion stock were removed at each time point as the digestion solution was agitated. Each aliquot was immediately quenched by neutralization with 15 µL of 0.2 M sodium carbonate per 50 µL of SGF [25]. Quenched samples were kept on ice until 2x SDS-PAGE loading buffer was added to each sample. Mixtures were then heated for 5 min in boiling water and stored at −20°C until analysis.

### Statistical analysis

Data analysis of intestinal worm burdens and fecal egg counts was carried out and plotted using Prism 5 (GraphPad Software Inc., La Jolla, CA, U.S.A.). For worm burdens, average indicates the average worm burdens amongst all the mice in each treatment group. For fecal egg counts, average indicates the egg count per mouse averaged from all mice in the group at a given time point. Fecal egg count data was analyzed via pair-wise comparisons between groups and days through two-way analysis of variance (ANOVA) with repeated measures and Bonferroni post tests. Results were as follows: F_Treatment_ = 70.69, degrees of freedom (df) = 1, P<0.0001; F_Time_ = 5.241, df = 3, P = 0.003; F_Interaction_ = 11.43, df = 1,3, P<0.0001. Worm burdens for Cry5B treatment versus placebo were compared using Mann-Whitney U test (one-tailed). Values are as follows: U = 7.5, P = 0.0007 for HD1 Cry5B versus placebo; U = 2.0, P = 0.0012 for 4Q7 Cry5B versus placebo. Worm burdens for tribendimidine experiment were compared using one-way ANOVA and Tukey's Multiple Comparison Test (F = 9.387, df = 3, 19, P = 0.0005).

## Results

### A single dose of Cry5B is able to achieve a significant therapeutic effect against *H. bakeri* parasites *in vivo*

To determine if Cry5B could provide therapy as a single-dose anthelmintic against *H. bakeri*, we treated *H. bakeri*-infected mice with Bt spore-crystal lysates expressing or not expressing (placebo control) Cry5B. When the bacterium Bt sporulates, it produces spores, large crystal protein-containing inclusions, and lysate produced when the mother cell that gives rise to the spore and crystal lyses upon completion of sporulation. Bt spore-crystal lysates (SCLs) from many Bt strains, including Bt *kurstaki* HD1 that targets caterpillars (Lepidoptera), have been extensively tested against mammals (including humans) and found to be non-pathogenic [15],[26],[27]. We transformed a crystal protein-minus HD1 strain with a Cry5B-expressing plasmid. Twenty mice were infected with *H. bakeri* larvae. Fifteen days post-infection (P.I.), we delivered into each mouse *per os* either a single 0.1 mL dose of Cry5B-containing HD1 SCLs (715 nmoles/kg or 100 mg/kg of Cry5B) or, as a placebo control, a single 0.1 mL dose of spore lysates (SLs, crystal-minus) from the parent, untransformed HD1 strain. Beginning the day before treatment (day 14 P.I. or day -1 treatment), and then continuing every other day (day 1, 3, 5 post-treatment), we collected fecal samples from each mouse to measure parasite progeny production (eggs/gram of feces). Five days after treatment (day 20 P.I.), the mice were euthanized and the total number of parasites present in the small intestine tallied.

**Figure 1:**
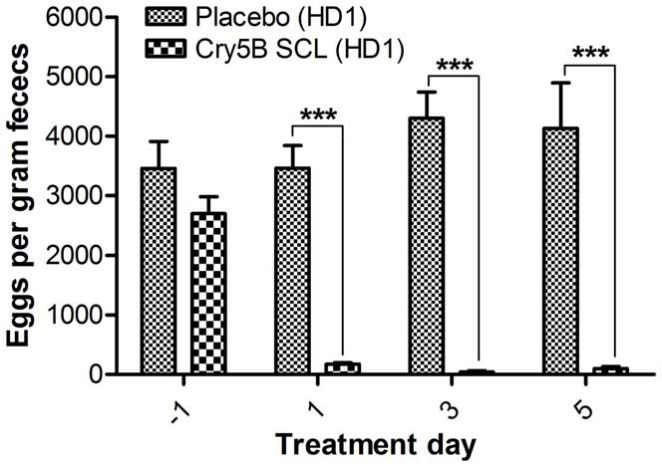
Effects of Cry5B on egg production in *Heligmosomoides bakeri* infected mice. Shown are the average eggs/gram of feces/mouse for both placebo (n = 10) and Cry5B HD1-(n = 10) treated groups the day before treatment (−1) and then every other day thereafter until the animals were euthanized on day 5 post-treatment.

**Figure 2:**
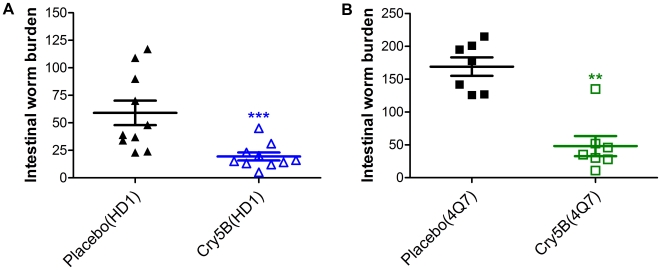
Effects of Cry5B on intestinal worm burdens in *Heligmosomoides bakeri* infected in mice. A. Shown are the intestinal worm burdens in placebo (HD1 SL) and Cry5B (HD1 SCL) treated mice (n = 10 each group). Infected mice were given a single dose of 715 nmoles/kg (100 mg/kg) Cry5B on day 15 P.I. and intestinal worm burdens assessed on day 20 P.I. The worm burdens in each mouse are indicated with a separate symbol. Long horizontal bars represent mean worm burdens; smaller bars indicate sem (standard error of the mean). B. Shown are the intestinal worm burdens in placebo (4Q7 SL) and Cry5B (4Q7 SCL) treated mice (n = 7 per group). Mice were given a single dose of 644 nmoles/kg (90 mg/kg) Cry5B on day 15 P.I. and intestinal worm burdens assessed on day 20 P.I. ** P<0.01, *** P<0.001.

With regards to progeny production, we found that on the day prior to treatment, the parasites in both groups of mice (placebo treated and Cry5B treated) were producing statistically indistinguishable amounts of eggs (Figure 1, Table 1). At days 1, 3, and 5 post-treatment, the placebo group showed no reduction in egg production, consistent with the hypothesis that the parent Bt strain alone has no effect on the parasites. In contrast, a rapid and remarkable reduction in egg production took place in the Cry5B-treated animals, resulting in a 95%, 99%, and 98% reduction on days 1, 3, and 5 post-treatment respectively (Figure 1, Table 1). With regards to parasite clearance, we found that the single dose of Cry5B achieved a remarkable therapeutic effect, clearing away 67% of the parasites relative to placebo control (Figure 2A, Table 2). Thus, a single dose of Cry5B has strong effects on parasite reproduction and the ability of parasites to maintain an infection.

**Table 1:** Fecal egg counts in Cry5B HD1 experiment.

	Day number relative to treatment			
	−1	1	3	5
Placebo	3455±457	3463±381	4303±439	4130±766
Cry5B (715 nM/kg)	2698±282	170±30	43±17	100±33
Reduction relative to placebo	N/A	95.1%	99.0%	97.6%

Numbers shown are eggs/gram of feces/mouse averaged for all 10 mice in each group ± standard error of the mean (sem).

**Table 2:** Worm burdens in Cry5B treatment experiments.

Group (no. of mice)		Intestinal worm burden (mean ± sem)	% Reduction relative to placebo
HD1	placebo (10)	59.1±11.1	NA
	Cry5B (10)	19.4±3.6	67.2
4Q7	placebo (7)	169.1±14.0	NA
	Cry5B (7)	48.1±15.3	71.5

placebo: crystal-deficient HD1 or 4Q7 strain; Cry5B: the corresponding strain transformed with a Cry5B-expressing plasmid. Cry5B dosage: 715 nm/kg (HD1) or 644 nm/kg (4Q7).

sem  =  standard error of the mean.

NA  =  not applicable.

The reduction in fecal egg count (>97%) was much larger than would be expected from the final mouse worm burden of the SCL-treated animals (67% cleared). There are at least two possible explanations for this—either the treatment was affecting the status of the worms so that any worms left behind were severely compromised in health or the treatment was preferentially eliminating female over male parasites. To distinguish between these possibilities, we made a note of the number of females present in placebo versus Cry5B treated controls during the counting of the worm burdens. In placebo treated mice we found that there were 35.3±5.9 (standard error of the mean, or sem) females while in the Cry5B treated mice there were 7.8±2.3 (sem) females per mouse intestine. Thus, there was a 78% reduction in the number of females present. This drop, although greater than that for males (51% reduction), does not seem sufficient to account for the observed >97% drop in egg production seen. These data suggest that the parasites that remained in the intestine were severely compromised in health.

We also determined if this capacity to clear an infection was dependent upon a particular Bt strain. We performed a similar curative experiment, measuring intestinal worm burdens after treatment, using the Bt strain 4Q7 (derived from Bt *israelensis*, which targets Diptera) either transformed with the Cry5B-expressing plasmid or untransformed. Fourteen mice were infected with *H. bakeri* larvae, and fifteen days P.I. a single dose of 0.1 mL Cry5B-containing 4Q7 SCLs or 0.1 mL 4Q7 SLs (crystal-minus) were delivered *per os*. The dose delivered per mouse was 644 nmoles/kg (90 mg/kg). We found a similar therapeutic effect as above—71% of the parasites were cleared relative to placebo control (Figure 2B, Table 2). We note that in this experiment the total number of parasites present in the small intestine in placebo control animals was greater than in the first experiment. The variability appears to be due to relative infectivity of different batches of L3 parasite larvae. These data demonstrate that, regardless of parent Bt strain and of the initial parasite load, a similar single dose of Cry5B is able to achieve comparable therapeutic effect.

### Cry5B compares highly favorably versus known anthelmintics

These results are significant when the relative efficacy of Cry5B is compared to other standard anthelmintic treatments. Published reports, employing a treatment timeline against *H. bakeri* parasites that is similar to our own, show that levamisole (10 mg/kg or 49 µmoles/kg delivered on day 12 P.I.) effected a 90% reduction in worm burdens and ivermectin (5 mg/kg or 5.7 µmoles/kg) or pyrantel (50 mg/kg or 84 µmoles/kg) or piperazine (4000 mg/kg or 46 mmoles/kg) delivered on day 18 P.I. effected an 87%, 98%, and 34% reduction in worm burdens respectively ([28],[29]. Another study showed that 2.9 µmoles/kg of ivermectin delivered on day 10 P.I. effected ∼70% reduction in *H. bakeri* worm burdens [30]. We could not find comparable studies with *H. bakeri* and benzimidazoles, although we did find that mebendazole delivered for 7 consecutive days, starting day 9 P.I. at 22 mg/kg/dose or 75 µmoles/kg/dose, achieved an 84% reduction in worm burdens [31]. Benzimidazoles (including albendazole) in general seem to be less active against *H. bakeri* [32]. Therefore, our single dose of ∼700 nmoles/kg (which is the highest dose we can currently pipette with SCLs) that achieved ∼70% reduction in worm burdens is 70X, 4–8X, 120X and 65,000X *lower* than the doses of levamisole, ivermectin, pyrantel, and piperazine used in the above studies.

**Figure 3:**
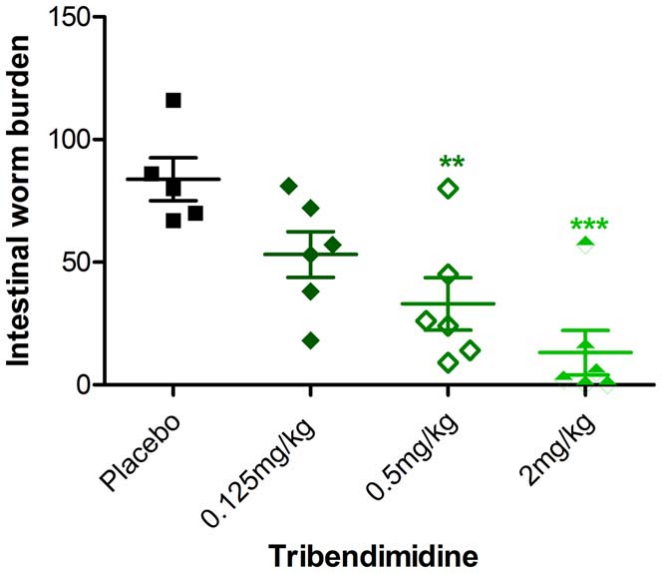
Effects of tribendimidine on *Heligmosomoides bakeri* infections in mice. Shown are the intestinal worm burdens in placebo (20 mM citrate buffer pH 7.3) and for various doses of tribendimidine. Data are plotted as in Figure 1. Infected mice (n = 6/group except n = 5 placebo) were given a single dose of tribendimidine on day 15 P.I. and intestinal worm burdens assessed on day 20 P.I. **P<0.01, ***P<0.001 relative to placebo control. Conversion to molar amounts is as follows: 0.125, 0.5, and 2 mg/kg are equivalent to 0.28, 1.1, and 4.4 µmoles/kg respectively.

This comparison suggests that the efficacy of Cry proteins relative to known anthelmintics is excellent. To directly compare our results to a known anthelmintic using the same treatment conditions, we performed curative experiments using the newest human anthelmintic and the only one taken to human clinical trials in the past thirty years, tribendimidine. We performed dose-dependent curative assays with tribendimidine against *H. bakeri* infections, finding an estimate dose of ∼1 mg/kg or 2.2 µmoles/kg tribendimidine to give a curative effect similar to ∼700 nmoles/kg Cry5B (Figure 3, Table 3). Based on this comparison, Cry5B is at least as good as tribendimidine at curing *H. bakeri* infections and in fact appears to be ∼2–3 fold superior.

**Table 3:** Worm burdens in tribendimidine treatment experiment.

Group (n = number of mice)	Intestinal worm burden (mean ± sem)	% Reduction relative to placebo
Placebo (n = 5)	83.8±8.7	N/A
0.125 mg/kg trib (n = 6)	53.2±9.3	36.5
0.5 mg/kg trib (n = 6)	33.0±10.7	60.6
2 mg/kg trib (n = 6)	13.4±9.1	84.0

sem  =  standard error of the mean.

N/A not applicable.

trib  =  tribendimidine.

### Cry5B is rapidly digested in simulated gastric fluids

These data indicate that Cry5B is an excellent anthelmintic when delivered at a single dose. However, Cry proteins are thought to be digested rapidly in the mammalian digestive tract, most notably by the acidic stomach [33]. If so, then it is possible that the dose of Cry protein reaching the parasites might have been very small.

**Figure 4:**
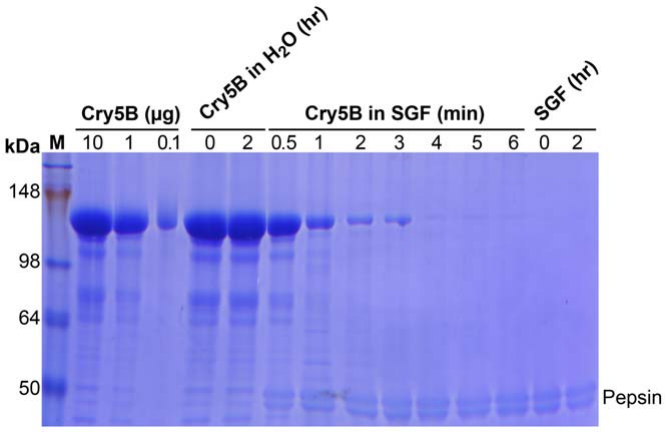
Behavior of Cry5B in simulated gastric fluids. Left lane, markers. Next three lanes: Cry5B loading control 10, 1, and 0.1 µg Cry5B in HD1 SCLs. Next two lanes: Cry5B (10 µg/lane) SCLs in water incubated for 0 or 2 h at 37°. No degradation is seen. Next seven lanes: Cry5B (10 µg/lane) SCLs incubated in simulated gastric fluid (SGF) for the time indicated (in minutes). Cry5B has nearly disappeared after four minutes. Right two lanes: simulated gastric fluids (no Cry5B) incubated for 0 and 2 h at 37° to demonstrate where pepsin runs on the gel. 8% SDS polyacrylamide gel stained with Coomassie blue.

To determine how well Cry5B would survive the mammalian stomach, we incubated Cry5B HD1-derived SCLs in simulated gastric fluids. We find that Cry5B is almost completely digested in this environment within four minutes (Figure 4). These data suggest that very little Cry5B is actually reaching the parasites.

## Discussion

Our study demonstrates that the Bt Cry protein Cry5B is an excellent anthelmintic *in vivo* against a natural and chronic intestinal roundworm infection in mice, namely *H. bakeri*. Cry5B is able to achieve significant reductions in parasite egg production (∼98%) and intestinal worm burdens (∼70%) following a single dose delivered *per os* at ∼700 nmoles/kg. This therapeutic effect, on a mole-by-mole basis, is on par with or superior to those of other anthelmintics commonly used in human therapy. Although this level of efficacy may seem surprising at first glance, upon deeper reflection it is not. Cry proteins, although they only attack the gut cells of invertebrates, are pore-forming toxins (PFTs; [34]). PFTs are the single most common virulence factors made by pathogenic bacteria and are also used by our immune system to combat pathogens [35],[36]. PFTs are potent weapons and the consequences of their attack on the integrity of the plasma membrane are great. In combination with previous data showing that Cry5B is also able to cure *A. ceylanicum* infections in hamsters [18], we have now demonstrated *in vivo* anthelmintic activity of Cry5B against two very different parasitic nematodes (one a blood feeder, the other not) in two different mammalian hosts. Taken together, along with the fact that Cry5B is active against *Nippostrongylus brasiliensis* larvae, against *Haemonchus contortus* larvae *in vitro*, against a phylogenetically wide range of free-living nematodes, and against the plant-parasitic nematode *Meloidogyne incognita* [16],[17],[37], our data indicate that Cry5B has very broad anti-nematode activity and that Cry5B has superb potential in human anthelmintic therapy.

As a natural product, it is interesting to compare the efficacy of Cry5B to other natural product anthelmintics. No recently investigated biological treatments against *H. bakeri* demonstrate comparable *in vivo* efficacy using single-dose regimens. Many of these natural compounds, such as the extract of *Embelia schimperi*, nitazoxanide, santonin, and *Myrsine Africana*, showed only small reductions in intestinal worm burden as a single dose, with efficacies of 30%, 21%, 18%, and 10%, respectively [29],[38],[39]. A single dose of 500 mg/kg of *Albizia anthelmintica*, not only revealed low efficacy, with a total worm burden reduction of only 3–23%, but also displayed significant toxicity [28]. Even the macrolactam *N*-methylfluvirucin, delivered at a daily dose of 50 mg/kg over 3 days, effected only a 42% reduction in total worm burden [40]. While other compounds were more efficient, they required extremely high doses and/or multiple-day dosing regimens. These included a daily treatment of ethanol extract of *Canthium manni* (Rubiaceae) at 5600 mg/kg, which showed a 75% decrease in fecal egg count and 84% reduction in worm burden with 7 days of treatment [31]. A 600 mg/kg treatment with extract of stem bark of *Sacoglottis gabonensis* was extremely effective, but exceedingly toxic, with mice showing signs such as depression, drowsiness, unsteady gait and paralysis of the hind limbs, dyspnoea, coma and death apparent within 1–2 min following intraperitoneal injection [41]. Perhaps the most promising of other natural treatments is papaya latex. A single-dose administration of papaya latex at 8 g/kg achieved an efficacy of 84.5%, with fecal egg count reductions of 93.3% [42]. Mice treated daily over 7 days with 133 nmoles of papaya latex showed a decrease in fecal egg count of 87–97% and a 92% reduction of worm burden [43]. In general, few of the natural compounds tested above proved to be practical treatments due to dosing and toxicity issues.

It is clear that Cry5B has great promise as an effective, safe, and much-needed addition to anthelmintic therapy. The vertebrate and human safety profiles of Cry proteins are outstanding—Cry proteins as insecticides are used around the world on a large-scale in organic farming, in aerial spray campaigns, and in vector (mosquito, black fly) control programs and have even been approved for expression in transgenic foods such as corn, potatoes, and rice [14],[44]. Although Cry5B has not been studied in this regard, it is a member of the same family of three-domain Cry proteins expressed in transgenic crops and used in all these spray programs and thus is predicted to have the same safety profile. Indeed, extensive research from our laboratory has confirmed that the receptor Cry5B needs to bind to in order to intoxicate nematodes is an invertebrate-specific glycan (carbohydrate) [45].

It is interesting to note that, although Cry5B has comparable if not superior activity against *H. bakeri* on a mole-by-mole basis with other anthelmintics, it is likely that only tiny amounts of the protein being delivered *per os* in our experiments are reaching the parasites. In four minutes, virtually all Cry5B is degraded in simulated gastric fluids. These experiments suggest that a simple enteric coating to protect Cry proteins against the stomach while releasing it in the small or large intestines might greatly increase the efficacy of Cry proteins. These data thus emphasize the importance of formulation in the next stage in the evolution of Cry protein anthelmintic development and suggest that such a formulation has the potential to reveal an anthelmintic with therapeutic properties comparable or superior to those currently in use.

## Supporting Information

Alternative Language Abstract S1 Translation of the abstract into Mandarin Chinese by YH.
(0.05 MB PDF)
