# Supplementary material for: Bacillus thuringiensis Cry5B Protein Is Highly Efficacious as a Single-Dose Therapy against an Intestinal Roundworm Infection in Mice
Source: PLoS Negl Trop Dis. 2010 Mar 2;4(3):e614. doi: 10.1371/journal.pntd.0000614 (PMC2830470; doi:10.1371/journal.pntd.0000614)
Supplement: Alternative Language Abstract S1 — Translation of the abstract into Mandarin Chinese by YH. (0.05 MB PDF) [file pntd.0000614.s001.pdf]

摘要：

研究背景： 肠道寄生线虫病是目前危害最为严重的疾病之一，全球感染的人群超过十亿并导致了严重的病理危害，尤其是对孩子和孕妇可引起很高的发病率。迄今，仅有一种具有一定疗效的药物，阿苯哒唑，用于肠道线虫感染的群体治疗，尽管三苯双脒可能作为第二种药物选择。鉴于大量人群需予治疗，受到寄生虫产生药物抗性的威胁以及现用（驱虫药物）疗效上的不足，故急需研制出新的（高效）驱虫药物。苏云金芽孢杆菌晶体蛋白是世界上最为常用的生物杀虫剂，并被认为对脊椎动物无毒性。

方法／主要研究成果：本文报道了线虫杀虫剂苏云金芽孢杆菌晶体蛋白Cry5B可治愈慢性和自然感染 *Heligmosomoides bakeri*的小鼠，该线虫早期亦被称为多形螺旋线虫 *(H.polygyrus)*。感染小鼠用两株不同的苏云金芽孢杆菌宿主菌所表达的约700 nmoles/kg (90-100mg/kg)Cry5B单剂量口服后，粪便虫卵减少98％，虫的负荷减少70％。其次，根据我们的数据和现有的文献研究结果的报道，Cry5B晶体蛋白对*Heligmosomoides bakeri* 感染的疗效，相当于或者优于现有抗蠕虫药物。再者，我们亦证实，Cry5B晶体蛋白在胃内非常迅速地被降解，故作用于该寄生虫的实际剂量是非常小的。

结论／意义：本研究结果表明，苏云金芽孢杆菌晶体蛋白Cry5B在体内具有很好的抗肠道线虫的作用。如果将该蛋白进行适当的制剂化处理，它将可能成为一种新型的高效的抗蠕虫药物。
